# Supplementary material for: Content-rich biological network constructed by mining PubMed abstracts
Source: BMC Bioinformatics. 2004 Oct 8;5:147. doi: 10.1186/1471-2105-5-147 (PMC528731; doi:10.1186/1471-2105-5-147)
Supplement: Additional File 2 — The original results of the above study (non-essential files are deleted to keep the file size under the limit set by BMC bioinformatics). [file 1471-2105-5-147-S2.bz2 › chilibotAdditionalFile2/dip50/729ID11333269E18086/html/DNAB_DNAC.html]

 


 **DNAB** and **DNAC** 
  
Found 151 abstracts in PubMed, retrieved 50.  
 

 What does Google say? 
 PDF only 
| .edu only 

---

**Interactive relationship** (e.g. stimulation, inhibition, etc)

**Neutral relationship**- The complex is arranged on the basis of interactions among  **DnaC**  and  **DnaB**  dimers.  Ref: 11250911 EMBO J, 2001
- **DnaC**  loads the  **DnaB**  helicase onto DNA as a prelude for primosome assembly and DNA replication.  Ref: 8722757 Genetics, 1996
- and e that  **DnaC**  interacts only with  **DnaB**  particles that have C 3 symmetry.  Ref: 11031115 J Mol Biol, 2000
- Interactions of the Escherichia coli  **DnaB**  Helicase Hexamer with the Replication Factor the  **DnaC**  Protein.  Ref: 12767828 J Mol Biol, 2003
- Quantitative studies of interactions between the Escherichia coli replication factor  **DnaC**  protein and the  **DnaB**  helicase have been performed using sedimentation velocity and fluorescence energy transfer techniques.  Ref: 12767828 J Mol Biol, 2003
- The replicative DNA helicase  [ **DNAC** ]   [ **DNAB** ]  encoded by the  **dnaB**  gene is essential for chromosomal DNA replication in Escherichia coli.  Ref: 7476872 Mol Gen Genet, 1995
- The  **DnaC**  protein is required for loading the  **DnaB**  helicase at oriC.  Ref: 8596432 Mol Microbiol, 1995
- We show that DnaI and  **DnaB**  interact physically and functionally with the  **DnaC**  helicase and mediate its functional delivery onto DNA.  Ref: 12718886 Mol Cell, 2003
- Escherichia coli  **DnaC**  protein bound to ATP forms a complex with  **DnaB**  protein.  Ref: 11333269 J Biol Chem, 2001
- Purified G35P binds single strand DNA ssDNA and double strand dsDNA and specifically interacts with SPP1 encoded replicative DNA helicase  [ **DNAC** ]   [ **DNAB** ]  G40P and SSB protein G36P.  Ref: 12124388 J Biol Chem, 2002
- In E.coli DnaT and  **DnaC**  are responsible for loading  **DnaB**  onto the DNA during primosome assembly, in the PriA and DnaA mediated cascades, respectively.  Ref: 11842108 Nucleic Acids Res, 2002
- Essential amino acids of Escherichia coli  **DnaC**  protein in an N terminal domain interact with  **DnaB**  helicase.  Ref: 11333269 J Biol Chem, 2001
- This novel switch in ssDNA binding activity evoked by complex formation suggests that interactions of P or  **DnaC**  with ssDNA may precede the transfer of  **DnaB**  onto DNA during initiation of DNA replication.  Ref: 9037022 Proc Natl Acad Sci U S A, 1997
- The six  **DnaC**  protomers bind tightly to the sixfold face of the  **DnaB**  hexamer.  Ref: 9562559 Structure, 1998
- The data establish that, in the presence of the ATP analog AMP PNP, or ADP, a maximum of six  **DnaC**  monomers bind cooperatively to the  **DnaB**  hexamer.  Ref: 12767828 J Mol Biol, 2003
- Thus, Cdc6 may act as the functional homologue of the bacterial  **DnaC**  that utilizes ATP hydrolysis to assemble the  **DnaB**  helicase at the origin.  Ref: 12837750 J Biol Chem< MedlineTA>J Biol Chem, 2003
- The bacteriophage lambda P and Escherichia coli  **DnaC**  proteins are known to recruit the bacterial  **DnaB**  replicative helicase to initiator complexes assembled at the phage and bacterial origins, respectively.  Ref: 9037022 Proc Natl Acad Sci U S A, 1997
- DNA replication in these mutants has a fast stop phenotype, indicating that the S. aureus  **dnaC**  gene encodes the replicative DNA helicase  [ **DNAC** ]   [ **DNAB** ]  required for the elongation step.  Ref: 12023093 FEMS Microbiol Lett, 2002
- A functional interaction between the putative primosomal protein DnaI and the main replicative DNA helicase  [ **DNAC** ]   [ **DNAB** ]   **DnaB**  in Bacillus.  Ref: 11842108 Nucleic Acids Res, 2002
- Experiments have been performed with the fluorescein modified  **DnaB**  helicase, which allows an exclusive monitoring of the  **DnaB**   **DnaC**  complex formation.  Ref: 12767828 J Mol Biol, 2003
- A protein encoded by the Staphylococcus aureus  **dnaC**  gene has 44% and 58% homology with Escherichia coli  **DnaB**  and Bacillus subtilis  **DnaC**  replicative DNA helicases, respectively.  Ref: 12023093 FEMS Microbiol Lett, 2002
- Primosome assembly proteins PriA, PriB, DnaT, and the  **DnaB**   **DnaC**  complex then promote the binding of the replicative helicase  **DnaB**  on the lagging strand template of the Mu fork.  Ref: 11459960 Proc Natl Acad Sci U S A, 2001
- In solution, the  **DnaC**  protein binding site is located on the large 33 kDa domain of the  **DnaB**  helicase.  Ref: 12767828 J Mol Biol, 2003
- The comparison of  **DnaC**  homologs of enterobacteria and enterobacterial phages supports the experimental data on residues important in interaction with  **DnaB** .  Ref: 12018708 Virus Genes, 2002
- These studies indicate that both P and  **DnaC**  contain a cryptic ssDNA binding activity that is mobilized when each forms a complex with the  **DnaB**  helicase.  Ref: 9037022 Proc Natl Acad Sci U S A, 1997
- **DnaB**  is delivered from this complex onto the DNA template, with the subsequent release of  **DnaC** .  Ref: 9562559 Structure, 1998
- These data suggest strongly that the  **DnaC**   **DnaB**  complex exists in vivo as a mixture of complexes with a different number of bound  **DnaC**  molecules, although the complex with six  **DnaC**  molecules bound dominates the distribution.  Ref: 12767828 J Mol Biol, 2003
- ... seven proteins, PriA, PriB, PriC, DnaT,  **DnaB** ,  **DnaC** , and DnaG, were required for primosome assembly on phi X174 DNA, only  **DnaB** ,  **DnaC** , and DnaG were required for replication from oriC, suggesting that the other proteins were not involved in chromosomal replication.  Ref: 10506828 Prog Nucleic Acid Res Mol Biol, 1999
- Thus  **DnaC**  promotes the formation of the pre replication complex, but must leave the complex in order for the  **DnaB**  protein to function as a helicase.  Ref: 8596432 Mol Microbiol, 1995
- We also present evidence that the  **DnaC**  helicase loading mechanism appears to be of the ring assembly type, proceeding through the recruitment of  **DnaC**  monomers and their hexamerization around single stranded DNA by the coordinated action of DnaI and  **DnaB** .  Ref: 12718886 Mol Cell, 2003
- The results show that  **DnaC**  interacts with replication forks during elongation in vivo, probably by binding to  **DnaB**  and inhibiting its helicase activity.  Ref: 8596432 Mol Microbiol, 1995
- Concomitantly, the capacity of  **DnaB**  to bind to ssDNA, as judged by UV crosslinking analysis, is suppressed upon formation of a P x  **DnaB**  or a  **DnaB**  x  **DnaC**  complex.  Ref: 9037022 Proc Natl Acad Sci U S A, 1997
- The analysis of  **dnaC**  alleles that preserved viability under elevated expression revealed an N terminal domain of  **DnaC**  involved in binding to  **DnaB** .  Ref: 11333269 J Biol Chem, 2001
- Cryptic single stranded DNA binding activities of the phage lambda P and Escherichia coli  **DnaC**  replication initiation proteins facilitate the transfer of E. coli  **DnaB**  helicase onto DNA.  Ref: 9037022 Proc Natl Acad Sci U S A, 1997
- In vitro, a slight excess of  **DnaC**  inhibits the movement of replication forks by inhibiting  **DnaB**  helicase activity Allen and Kornberg, 1991.  Ref: 8596432 Mol Microbiol, 1995

**Non-interactive relationship** (e.g. studied together, co-existance, homology, etc.)

- These mutations are alleles of genes 32 and 41, which respectively encode a single stranded DNA binding protein gp32 and the replicative DNA helicase  [ **DNAC** ]   [ **DNAB** ]  gp41.  Ref: 12697750 J Biol Chem< MedlineTA>J Biol Chem, 1995
- The significance of the results in the functioning of the  **DnaB**  helicase  **DnaC**  protein complex is discussed.  Ref: 12767828 J Mol Biol, 2003
- CONCLUSIONS The structures of the  **DnaB**  helicase and its complex with  **DnaC**  reveal some interesting structural features relevant to helicase function and to the assembly of the two protein complex.  Ref: 9562559 Structure, 1998
- **DnaB**  is the major replicative DNA helicase  [ **DNAC** ]   [ **DNAB** ]  in Escherichia coli and has been used as a model system for studying the structure and function of hexameric helicases.  Ref: 9562559 Structure, 1998
- In contrast, in Bacillus subtilis, in addition to the  **DnaB**  counterpart called  **DnaC** , we have found a second essential DNA helicase, called PcrA.  Ref: 9701819 Mol Microbiol, 1998
- d that the  **DnaB** . **DnaC**  complex exists only as particles with C 3 symmetry.  Ref: 11031115 J Mol Biol, 2000
- Here, we measure the translocation and energy coupling efficiency of a replicative DNA helicase  [ **DNAC** ]   [ **DNAB** ]  from bacteriophage T7 that is a member of a class of helicases that assembles into ring shaped hexamers.  Ref: 12206763 J Mol Biol, 2002
- **DnaC**  is also involved, because it must be in a complex with  **DnaB**  for delivery of the helicase.  Ref: 12161435 J Biol Chem, 2002
- It is therefore likely that an MCM complex acts as the replicative DNA helicase  [ **DNAC** ]   [ **DNAB** ]  in eukaryotes and archaea.  Ref: 10677495 Proc Natl Acad Sci U S A, 2000
- Co overexpression of  **DnaB**  protein alleviated the inhibition of replication caused by moderate excess of  **DnaC** .  Ref: 8596432 Mol Microbiol, 1995
- We also propose from the phylogenetic analysis of phage encoded homologs of E. coli  **DnaB**  and  **DnaC** , that the recombination with the host sequences is not frequent.  Ref: 12018708 Virus Genes, 2002
- Subsequent formation of the preprimosome involves the ATP dependent transfer of  **DnaB**  from a  **DnaB**   **DnaC**  complex to the PriA PriB DnaT PAS complex.  Ref: 8663104 J Biol Chem, 1996
- The T4 gp59 protein is the major accessory protein of the phage s replicative DNA helicase  [ **DNAC** ]   [ **DNAB** ] , gp41.  Ref: 10428868 J Biol Chem, 1999
- We propose that the tight MCM Cdc45 complex functions as a replicative DNA helicase  [ **DNAC** ]   [ **DNAB** ]  in vivo.  Ref: 12581157 Genes Cells, 2003
- In Escherichia coli, the primosome assembly proteins, PriA, PriB, PriC, DnaT,  **DnaC** ,  **DnaB** , and DnaG, are thought to help to restart DNA replication forks at recombinational intermediates.  Ref: 10835375 Genetics, 2000
- The gene 59 protein gp59 of bacteriophage T4 stimulates the activities of gene 41 protein gp41, the T4 replicative DNA helicase  [ **DNAC** ]   [ **DNAB** ] , by promoting the assembly of gp41 onto single stranded ss DNA molecules that are covered with cooperatively bound gene 32 protein gp32.  Ref: 8702746 J Biol Chem, 1996
- Upon promoting transfer of Mu ends to target DNA, the Mu transpososome undergoes transition to a pre replisome that permits initiation of DNA synthesis only in the presence of primosome assembly proteins PriA, DnaT,  **DnaB**  and  **DnaC** .  Ref: 9362501 EMBO J, 1997
- This dual switching process may underlie the sensitivity of  **DnaB**  to even small fluctuations in  **DnaC**  levels.  Ref: 12065427 EMBO J, 2002
- SPP1 encoded replicative DNA helicase  [ **DNAC** ]   [ **DNAB** ]  gene 40 product G40P is an essential product for phage replication.  Ref: 12034814 Nucleic Acids Res, 2002
- We present the 26 A resolution three dimensional structure of the  **DnaB**  hexamer in complex with its loading partner,  **DnaC** , obtained from cryo electron microscopy.  Ref: 11250911 EMBO J, 2001
- To identify the domain of  **DnaC**  that interacts with  **DnaB** , a genetic selection was used based on the lethal effect of induced  **dnaC**  expression and a model that inviability arises by the binding of  **DnaC**  to  **DnaB**  to inhibit replication fork movement.  Ref: 11333269 J Biol Chem, 2001
- The speed of the Escherichia coli fork in vivo depends on the  **DnaB**   **DnaC**  ratio.  Ref: 8596432 Mol Microbiol, 1995
- This can be attributed to its ability, unlike wild type  **DnaC** , to catalyze a SSB insensitive general priming reaction with  **DnaB**  and DnaG on any SSB coated single stranded DNA.  Ref: 10713144 J Biol Chem, 2000
- Bacillus subtilis tau subunit of DNA polymerase III interacts with bacteriophage SPP1 replicative DNA helicase  [ **DNAC** ]   [ **DNAB** ]  G40P.  Ref: 12466528 Nucleic Acids Res, 2002
- Although, interaction of DnaA with  **DnaB**  could not substitute for RepA  **DnaB**  interaction for helicase loading, DnaA along with integration host factor,  **DnaC** , and RepA was essential for helicase loading.  Ref: 9874774 Proc Natl Acad Sci U S A, 1999
- We report the biochemical characterization of the bacterial hexameric replicative helicase  **DnaC**  of Bacillus subtilis with that of the two replication initiation proteins DnaI and  **DnaB** .  Ref: 12718886 Mol Cell, 2003
- However, in the presence of the DNA, there is a significant difference in the energetics and structure of the ternary complex,  **DnaC**   **DnaB**  ssDNA, formed in the presence of AMP PNP as compared to ADP.  Ref: 12767828 J Mol Biol, 2003
- The final preprimosomal complex contains PriA, PriB, DnaT, and  **DnaB**  but not  **DnaC** .  Ref: 8663104 J Biol Chem, 1996
- Three dimensional reconstructions from cryoelectron microscopy images reveal an intimate complex between helicase  **DnaB**  and its loading partner  **DnaC** .  Ref: 9562559 Structure, 1998
- As the three dimensional structure of the hexameric replicative DNA helicase  [ **DNAC** ]   [ **DNAB** ]  RepA encoded by the broad host range plasmid RSF1010 is known, this protein served as a model helicase to search for inhibitory compounds.  Ref: 11812837 Nucleic Acids Res, 2001
- Therefore, the ratio of  **DnaC**  to  **DnaB**  and the affinity of  **DnaC**  for a helicase hexamer at an established replication fork are of great importance for the rate of replication fork movement also in vivo.  Ref: 8596432 Mol Microbiol, 1995
- Other selected mutations that map to regions carrying Walker A and B boxes are expected to be defective in ATP binding, a required step in  **DnaB**   **DnaC**  complex formation.  Ref: 11333269 J Biol Chem, 2001
- Bacteriophage P2 requires several host proteins for lytic replication, including helicase  **DnaB**  but not the helicase loader,  **DnaC** .  Ref: 10756017 J Virol, 2000
- In this report, we determined that one  **DnaB**  helicase or one  **DnaB**   **DnaC**  complex is bound to a single stranded DNA in a biologically relevant DNA replication system.  Ref: 12161435 J Biol Chem, 2002
- We report here the structures of the  **DnaB**  helicase hexamer and its complex with  **DnaC**  under a defined set of experimental conditions, as determined by three dimensional cryoelectron microscopy.  Ref: 9562559 Structure, 1998
- The MCM2 7 complex is believed to function as the eukaryotic replicative DNA helicase  [ **DNAC** ]   [ **DNAB** ] .  Ref: 12087101 J Biol Chem, 2002
- The topology of the ternary complex  **DnaC**   **DnaB**  ssDNA has been determined using the fluorescence energy transfer method.  Ref: 12767828 J Mol Biol, 2003
- Transformation studies indicated that ORF2 consisting of a primase fused to a replicative DNA helicase  [ **DNAC** ]   [ **DNAB** ] , ori, and ORF3 constitute the minimal requirements for replication of pN42 in the heterologous host Lactococcus lactis.  Ref: 11982337 Plasmid, 2002
- The replication restart proteins, PriA, PriB, PriC,  **DnaB** ,  **DnaC** , DnaG, DnaT and Rep, form redundant pathways that recognize repaired replication forks and restart them.  Ref: 11532137 Mol Microbiol, 2001
- The  **DnaB** . **DnaC**  complex a structure based on dimers assembled around an occluded channel.  Ref: 11250911 EMBO J, 2001
- The RepA protein of plasmid RSF1010 is a replicative DNA helicase  [ **DNAC** ]   [ **DNAB** ] .  Ref: 9374507 J Biol Chem, 1997
- An S. pombe homolog of the replicative DNA helicase  [ **DNAC** ]   [ **DNAB** ]  DNA2 of S. cerevisiae suppresses cdc24.  Ref: 9649516 Genetics, 1998
- Although the DnaA protein directs the  **DnaB**   **DnaC**  complex to the plasmid replication origin, it cannot by itself activate the helicase.  Ref: 9407123 J Biol Chem, 1997
- Central to the process of primosomal assembly is the loading of the main hexameric replicative helicase  **DnaB**  in E.coli and  **DnaC**  in Bacillus subtilis on the DNA.  Ref: 11842108 Nucleic Acids Res, 2002
- Two cofactors, ssDNA and  **DnaB** , trigger hydrolysis of ATP, converting  **DnaC**  to the ADP form that no longer inhibits  **DnaB** .  Ref: 12065427 EMBO J, 2002
- A two protein strategy for the functional loading of a cellular replicative DNA helicase  [ **DNAC** ]   [ **DNAB** ] .  Ref: 12718886 Mol Cell, 2003
- This is based on the competition between the lambda P protein and the host DnaA and  **DnaC**  proteins for  **DnaB**  helicase.  Ref: 11429468 Microbiology, 2001
- Gel filtration chromatography was used to isolate both preprimosomal and primosomal complexes formed on single stranded DNA binding protein coated phiX174 DNA by the combination of PriA, PriB, PriC, DnaT,  **DnaB** ,  **DnaC** , and DnaG.  Ref: 8663105 J Biol Chem, 1996
- The gene 59 protein gp59 of bacteriophage T4 is an important accessory protein of the phage encoded replicative DNA helicase  [ **DNAC** ]   [ **DNAB** ] , gp41.  Ref: 9325092 J Mol Biol, 1997
- The initiation of replication from oriV RSF1010, the replication origin of the broad host range plasmid RSF1010, depends on RepA helicase, RepB primase, and RepC initiator protein, encoded by RSF1010 itself, while this initiation event in E. coli is independent of dnaA,  **dnaB** ,  **dnaC** , and dnaG Scherzinger et al.  Ref: 8690079 FEBS Lett, 1996
